# Delineating the genetic heterogeneity of OCA in Hungarian patients

**DOI:** 10.1186/s40001-017-0262-0

**Published:** 2017-06-19

**Authors:** Beáta Fábos, Katalin Farkas, Lola Tóth, Adrienn Sulák, Kornélia Tripolszki, Mariann Tihanyi, Réka Németh, Krisztina Vas, Zsanett Csoma, Lajos Kemény, Márta Széll, Nikoletta Nagy

**Affiliations:** 1 Mór Kaposi Teaching Hospital of the Somogy County, Kaposvár, Hungary; 2 MTA-SZTE Dermatological Research Group, University of Szeged, Szeged, Hungary; 3 Department of Medical Genetics, University of Szeged, 4 Somogyi Bela Street, 6720 Szeged, Hungary; 4 Genetic Laboratory, Hospital of Zala County, Zalaegerszeg, Hungary; 5 Department of Dermatology and Allergology, University of Szeged, Szeged, Hungary

**Keywords:** Oculocutaneous albinism, Concomitant analysis, *TYR* gene, *SLC45A2* gene, *OCA2* gene

## Abstract

**Background:**

Oculocutaneous albinism (OCA) is a clinically and genetically heterogenic group of pigmentation abnormalities characterized by variable hair, skin, and ocular hypopigmentation. Six known genes and a locus on human chromosome 4q24 have been implicated in the etiology of isolated OCA forms (OCA 1–7).

**Methods:**

The most frequent OCA types among Caucasians are OCA1, OCA2, and OCA4. We aimed to investigate genes responsible for the development of these OCA forms in Hungarian OCA patients (*n* = 13). Mutation screening and polymorphism analysis were performed by direct sequencing on *TYR*, *OCA2*, *SLC45A2* genes.

**Results:**

Although the clinical features of the investigated Hungarian OCA patients were identical, the molecular genetic data suggested OCA1 subtype in eight cases and OCA4 subtype in two cases. The molecular diagnosis was not clearly identifiable in three cases. In four patients, two different heterozygous known pathogenic or predicted to be pathogenic mutations were present. Seven patients had only one pathogenic mutation, which was associated with non-pathogenic variants in six cases. In two patients no pathogenic mutation was identified.

**Conclusions:**

Our results suggest that the concomitant screening of the non-pathogenic variants—which alone do not cause the development of OCA, but might have clinical significance in association with a pathogenic variant—is important. Our results also show significant variation in the disease spectrum compared to other populations. These data also confirm that the concomitant analysis of OCA genes is critical, providing new insights to the phenotypic diversity of OCA and expanding the mutation spectrum of OCA genes in Hungarian patients.

## Background

Oculocutaneous albinism (OCA) is a clinically and genetically heterogenic group of rare monogenic diseases characterized by diffuse reduced melanin production in the skin, hair, and/or eyes [1]. Eye symptoms including photophobia, nystagmus, strabismus, foveal hypoplasia, reduced iris, and retinal pigmentation and reduction in visual acuity are present in all types of albinism. To date, six genes and a locus on 4q24 human chromosomal region have been implicated in the development of the isolated OCA forms (OCA 1–7) [2]. Tyrosinase gene (*TYR*; OMIM 606933) is responsible for the development of OCA type 1 (OCA1) [3, 4]. Mutations of the oculocutaneous albinism two gene (*OCA2;* OMIM 611409) are associated with OCA type 2 (OCA2) [5]. Pathogenic variants of the tyrosinase-related protein gene (*TYRP*; OMIM 115501) are linked with OCA type 3 (OCA3) [6]. Mutations in a membrane-associated transporter gene (*SLC45A2*; OMIM 606202) are implicated in OCA type 4 (OCA4) [7]. OCA5 phenotype is linked to an unknown gene on human chromosome 4q24 [8]. Mutations of the sodium/calcium/potassium exchanger 5 gene (*SLC24A5*; OMIM 609802) encoding a solute carrier protein are associated with a new form of OCA, named as OCA6 [9]. The mutations of chromosome 10 open reading frame 11 gene (*C10ORF11*; OMIM 614537) are responsible for OCA7 type of albinism [10]. Furthermore, an additional 10 genes have been associated with syndromic OCA variants such as Hermansky–Pudlak syndrome (HPS) and Chediak–Higashi syndrome (CHS) [2]. Noted, there is a form of albinism, ocular albinism (OA1), affecting the eyes, but does not affect the hair and skin, which is caused by the mutation in G protein-coupled receptor 143 gene (*GPR143*; OMIM 300808) [11].

Oculocutaneous albinism affects approximately one in 20,000 individuals worldwide; however, the prevalence of its subtypes varies among different populations [12]. Although OCA1, OCA2, and OCA4 are present in Caucasian populations, the most common form is OCA1 [6]. OCA3 is present in mainly Africans and is rarely seen in other populations [6], OCA5 has been found in one Pakistani family to date [8], OCA6 is recently discovered in one Chinese family [9] and OCA7 has been explored in several Faroese families (Denmark) [10].

Since the most frequent forms of OCA in Caucasian population are OCA1, OCA2, and OCA4, we performed mutation screening of the *TYR*, *OCA2*, and *SLC45A2* genes to promote the understanding of disease heterogeneity, to assess the independent and cumulative contributions of these three genes to the disease development, and to compare relative and cumulative frequencies of disease variants in a representative Hungarian OCA population.

## Patients and methods

### Examined individuals

The individuals (*n* = 13) participating in this study were recruited at the Mór Kaposi Teaching Hospital of the Somogy County (Kaposvár, Hungary), at the Hospital of Zala County (Zalaegerszeg, Hungary) and at the Department of Dermatology and Allergology, University of Szeged (Szeged, Hungary). In the enrolled patients, the diagnosis of OCA was established in the presence of skin and hair hypopigmentation and distinctive ocular changes such as nystagmus, reduced iris pigmentation, reduced retinal pigmentation, and foveal hypoplasia (Table 1). All investigated individuals were Hungarians.

**Table 1:** Clinical features of the Hungarian OCA patients

Patient	Gender	Age	Skin	Hair color	Iris color
1	Male	75	Hypopigmented, no tanning ability	Snow-white	Blue
2	Male	7	Hypopigmented, no tanning ability	Snow-white	Blue
3	Female	57	Hypopigmented, no tanning ability	Snow-white	Blue
4	Male	48	Hypopigmented, no tanning ability	Snow-white	Blue
5	Female	60	Hypopigmented, no tanning ability	Snow-white	Blue
6	Male	11	Hypopigmented, no tanning ability	Snow-white	Blue
7	Male	15	Hypopigmented, no tanning ability	Snow-white	Blue
8	Female	3	Hypopigmented, no tanning ability	Snow-white	Blue
9	Female	31	Hypopigmented, no tanning ability	Snow-white	Blue
10	Female	28	Hypopigmented, no tanning ability	Snow-white	Blue
11	Male	21	Hypopigmented, no tanning ability	Snow-white	Blue
12	Male	6	Hypopigmented, no tanning ability	Snow-white	Blue
13	Male	4	Hypopigmented, no tanning ability	Snow-white	Blue

The investigation was approved by the Internal Ethical Review Board of the University of Szeged. Written informed consent was obtained from the patients and the healthy controls, and the study was conducted according to the Principles of the Declaration of Helsinki.

### Genetic investigation

Blood was drawn from the enrolled individuals, and genomic DNA was isolated using a BioRobot EZ1 DSP Workstation (QIAGEN; Godollo, Hungary). The entire coding regions and the flanking introns of the *TYR*, *OCA2*, and *SLC45A2* genes were amplified (primer sequences used were taken from the UCSC Genome Browser). Since a pseudogene of *TYR*, tyrosinase-like gene (*TYRL*; OMIM 191270) is known, which shows 98.55% identity with the 3′region of *TYR* (exon 4 and 5), specific primers were used for amplification of these regions [13]. Direct sequencing of PCR products was performed on an ABI 3100 sequencer and compared with the wild-type gene sequences using the Ensemble Genome Browser.

### Pathogenicity predictions

As in previous study [14], in silico tools were applied to identify the functional impact of the newly detected missense mutations. Here we used SIFT (Sorting Intolerant from Tolerant, PolyPhen 2.0 (Polymorphism Phenotyping), Mutation Taster, PROVEAN (Protein Variation Effect Analyzer) and PANTHER (Protein ANalysis THrough Evolutionary Relationships) tools. SIFT is based on the evolutionary conservation and predicts whether an amino acid substitution affects protein function. SIFT prediction score ranges from 0 to 1, and the amino acid substitution is predicted damaging if the score is less than an equal to 0.05, and tolerated if the score is greater than 0.05 [15]. PolyPhen 2.0 is based on structural and comparative evolutionary considerations and predicts the possible impact of an amino acid substitution on the stability and function of a protein. PolyPhen 2.0 uses the same range than SIFT (0–1) and the substitution is predicted to be possibly/probably damaging at greater than an equal to 0.5 value [16]. Mutation Taster is a prediction software based on the physicochemical properties of amino acids and scores substitutions according to the degree of difference between the original and the new amino acid (0–215) [17]. PROVEAN prediction is based on the sequence homology. If the PROVEAN score is equal to or below the default score threshold (−2.5), the protein variant is predicted to be deleterious and if the score is above the threshold, the variant is predicted to be neutral [18]. PANTHER program is a library of protein family and subfamily, which predicts the occurrence frequency of an amino acid in evolutionary conserved protein sequences. If the score is −3 or less, the variant is predicted has deleterious effect [19].

## Results

During the investigation of *TYR*, *OCA2*, and *SLC45A2* genes, we have identified pathogenic mutations in 84% (*n* = 11) of the examined individuals (*n* = 13), as shown in Table 2. In 4 cases, two heterozygous mutations have been found, suggesting a compound heterozygous state. Seven patients carried only one disease-causing mutation. Furthermore, in 11 cases out of 13, we have detected one or more common polymorphisms.

**Table 2:** Detected *TYR*, *SLC45A2*, and *OCA2* mutations and polymorphisms

Patient	Mutation 1	Mutation 2	Polymorphisms	Molecular diagnosis
1	*TYR* gene: c.74dupT p.Ser26Leufs*2 (hetero)	*TYR* gene: c.346C>T p.Arg116* (hetero)	–	OCA1
2	*TYR* gene: c.1037−7T>A (homo)	–	*SLC45A2* gene: c.1122G>C p.Leu374Phe (homo) Global MAF: 0.2750 Caucasian MAF: 0.0616 *OCA2* gene: c.913C>T p.Arg305Trp (hetero) Global MAF: 0.0790 Caucasian MAF: 0.0650	OCA1
3	*TYR* gene: c.1037−7T>A (homo)	–	*SLC45A2* gene: c.1122G>C p.Leu374Phe (homo) Global MAF: 0.2750 Caucasian MAF: 0.0616	OCA1
4	*TYR* gene: c.1037−7T>A (homo)	–	*SLC45A2* gene: c.1122G>C p.Leu374Phe (homo) Global MAF: 0.2750 Caucasian MAF: 0.0616	OCA1
5	*TYR* gene: c.1204C>T p.Arg402* (hetero)	–	*TYR* gene: c.575C>A p.Ser192Tyr (homo) Global MAF: 0.1234 Caucasian MAF: 0.3718 *SLC45A2* gene: c.1122G>C p.Leu374Phe (homo) Global MAF: 0.2750 Caucasian MAF: 0.0616	OCA1
6	*TYR* gene: c.650G>A p.Arg217Gln (hetero)	–	*TYR* gene: c.1205G>A p.Arg402Gln (hetero) Global MAF: 0.0813 Caucasian MAF: 0.2525 *SLC45A2* gene: c.1122G>C p.Leu374Phe (homo) Global MAF: 0.2750 Caucasian MAF: 0.0616	OCA1
7	*TYR* gene: c.650G>A p.Arg217Gln (hetero)	–	*TYR* gene: c.1205G>A p.Arg402Gln (hetero) Global MAF: 0.0813 Caucasian MAF: 0.2525 *SLC45A2* gene: c.1122G>C p.Leu374Phe (homo) Global MAF: 0.2750 Caucasian MAF: 0.0616	OCA1
8	*TYR* gene: c.650G>A p.Arg217Gln (hetero)	–	–	OCA1
9	*SLC45A2* gene: c.1226G>A p.Gly409Asp (hetero)	*SLC45A2* gene: c.1459C>T p.Gln487* (hetero)	*TYR* gene: c.575C>A p.Ser192Tyr (homo) Global MAF: 0.1234 Caucasian MAF: 0.3718 *TYR* gene: c.1205G>A p.Arg402Gln (hetero) Global MAF: 0.0813 Caucasian MAF: 0.2525	OCA4
10	*SLC45A2* gene: c.1226G>A p.Gly409Asp (hetero)	*SLC45A2* gene: c.1459C>T p.Gln487* (hetero)	*TYR* gene: c.575C>A p.Ser192Tyr (hetero) Global MAF: 0.1234 Caucasian MAF: 0.3718	OCA4
11	TYR gene: c.1366+4A>G (hetero)	*SLC45A2* gene: c.1099G>A p.Val367Ile (hetero)	*TYR* gene: c.575C>A p.Ser192Tyr (hetero) Global MAF: 0.1234 Caucasian MAF: 0.3718 *SLC45A2* gene: c.1122G>C p.Leu374Phe (homo) Global MAF: 0.2750 Caucasian MAF: 0.0616	OCA1/ OCA4
12	–	–	*TYR* gene: c.575C>A p.Ser192Tyr (homo) Global MAF: 0.1234 Caucasian MAF: 0.3718 *TYR* gene: c.1205G>A p.Arg402Gln (hetero) Global MAF: 0.0813 Caucasian MAF: 0.2525 *SLC45A2* gene: c.1122G>C p.Leu374Phe (homo) Global MAF: 0.2750 Caucasian MAF: 0.0616	Unknown
13	–	–	*SLC45A2* gene: c.1122G>C p.Leu374Phe (homo) Global MAF: 0.2750 Caucasian MAF: 0.0616	Unknown

*MAF* minor allele frequency

**Fig. 1:**
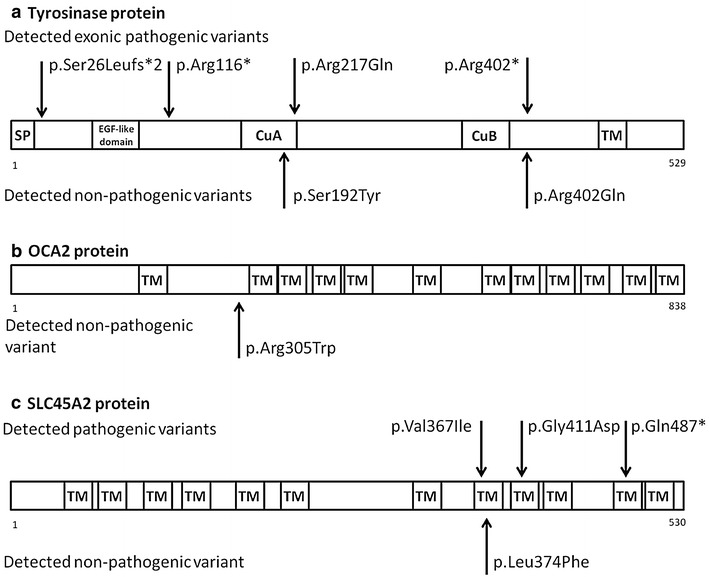
**a** Distribution of the detected *TYR* variants on the tyrosinase protein, **b** *OCA2* variant on the transporter protein, and **c** *SLC45A2* variants on the transporter protein (*SP* signal peptide, *EGF-like domain* epidermal-growth-factor-like domain, *CuA* copper-binding domain, *CuB* copper-binding domain, *TM* transmembrane domain)

Direct sequencing of the *TYR* gene revealed pathogenic mutations in 69% (*n* = 9) of the investigated patients. Only one patient carried two heterozygous mutations, a Thymin-base duplication (c.74dupT, p.Ser26Leufs*2) and a nonsense (c.346C>T, p.Arg116*) mutation in the first exon of *TYR* gene, suggesting a compound heterozygous state. Three patients carried the c.1037−7T>A splice site mutation in homozygous form. Out of these three patients, two (Patient 2 and 3) are related to each other and one (Patient 4) is not aware of any relationship with the other two mutation carriers. We have detected the c.1204C>T p.Arg402* nonsense mutation heterozygously in one case and the c.650G>A p.Arg217Gln missense mutation heterozygously in three patients. The heterozygous c.1366+4A>G splice site mutation has been detected in one patient. This patient was additionally heterozygous for the *SLC45A2* c.1099G>A p.Val367Ile mutation. Considering the common polymorphisms of *TYR* gene, the c.575C>A p.Ser192Tyr, and c.1205G>A p.Arg402Gln were detected in seven patients. The p.Ser192Tyr variant affects a copper-binding domain of the protein; all the other exonic pathogenic and non-pathogenic variants are located outside of the known functional domains of the enzyme (Fig. 1a).

No pathogenic *OCA2* mutation was identified in the investigated OCA individuals. However, one patient with the pathogenic c.1037−7T>A *TYR* mutation carried the common c.913C>T p.Arg305Trp polymorphism of the *OCA2* gene in heterozygous form. This variant does not affect any known functional domains of the OCA2 protein (Fig. 1b).

Based on our results, 3 of 13 patients carried *SLC45A2* pathogenic mutation. Two patients carried the combination of two novel mutation previously described by our workgroup: the c.1226G>A p.Gly409Asp missense mutation and the c.1459C>T p.Gln487* nonsense mutation. These mutations are not present in any SNP database (ExAC, 1000 Genome Project, ClinVar). Prediction analyses were performed to identify the functional role of the missense mutation. All prediction software suggested that the p.Gly409Asp mutation is deleterious (SIFT score: 0.002, damaging; PolyPhen 2.0 score: 0.996, probably damaging; Mutation Taster score: 94, disease causing; PROVEAN score: −3.25, deleterious; PANTHER score: −4.26, deleterious). The nonsense mutation was deemed to be pathogenic. One patient carried the c.1099G>A p.Val367Ile missense mutation beside the c.1366+4A>G splice site mutation on the *TYR* gene. In eight patients, only the non-pathogenic c.1122G>C p.Leu374Phe missense polymorphism was detected. All the detected pathogenic and non-pathogenic variants are located within transmembrane domains of the encoded protein (Fig. 1c).

## Discussion

This study reports the concomitant investigation of three genes (*TYR*, *OCA2*, and *SLC45A2*) in 13 Hungarian OCA patients, which have been implicated in the development of isolated OCA forms. The *TYR* gene encodes the tyrosinase enzyme, which catalyzes the first and second steps in melanin synthesis: the hydroxylation of tyrosine to l-DOPA and the oxidation of l-DOPA to DOPA-quinone [3]. The *OCA2* and *SLC45A2* genes encode transporter proteins, which are implicated in the trafficking of tyrosinase to melanosomes [20, 21].

Pathogenic *TYR* mutations were present in 69% (*n* = 9) of the patients. However, the sample size of this study is small, our results correlate well with previous findings that OCA1 is the most common isolated OCA subtype and *TYR* mutations account for approximately 25–50% of the isolated OCA cases worldwide [6, 22].

In our study, three missense variants were detected for the *TYR* gene. One of these variants is considered pathogenic (p.Arg217Gln) and two (p.Ser192Tyr, p.Arg402Gln) are considered common polymorphisms. The p.Arg217Gln mutation is located in a non-conservative region of tyrosinase protein, at this amino acid position two other known missense mutations are described (p.Arg217Gly, p.Arg217Trp) [23]. The polymorphisms of *TYR* gene were not directly related to pigmentation phenotypes in normal Caucasians, but their impact should be taken into account as an important modifier of human skin, hair, and eye color [24]. Functional studies reported that 192Tyr allele reduced tyrosinase activity, significant reduction in heterozygous and consistent decrease in homozygous form were observed, and the presence of 402Gln allele resulted significantly less TYR protein, displayed altered trafficking and glycosylation, with reduced DOPA oxidase [24]. The p.Arg402Gln polymorphism exhibits reduced tyrosinase activity at physiological temperature and is considered a temperature-sensitive variant [25–27]. This variant alone is unable to cause OCA; however, its increased frequency in OCA patients with one heterozygous pathogenic *TYR* mutation suggests that it can contribute to the development of OCA in combination with a pathogenic mutation [28]. Two Hungarian patients carried the p.Arg402Gln polymorphism in combination with the p.Arg217Gln pathogenic variant. Previous report suggests this combination might contribute to the development of the OCA symptoms of the patient [26].

In one case, compound heterozygosity for two mutations was found. A heterozygous T-base duplication (c.74dupT p.Ser26Leufs*2) and a nonsense mutation (c.346C>T, p.Arg116*) were identified on *TYR* gene. Both mutations lead to the development of a premature termination codon. Due to these changes, the translated mutant TYR protein is highly truncated and we assume that these enormous truncations of the mutant TYR protein may lead to its dysfunction.

Two of the nine Hungarian OCA patients with *TYR* mutations carry a combination of two pathogenic mutations. In three of nine, a splice site mutation was identified in homozygous form. In four of nine, only one heterozygous pathogenic mutation was identified. These results correlate well with the recently reported investigation of an Iranian OCA population: pathogenic *TYR* variants were identified in 19 of 30 patients, and in this study, six patients carried only one pathogenic *TYR* mutation, and any pathogenic mutation were not identified in five patients [4].

The pathogenic *TYR* mutations detected in the Hungarian OCA patients have been previously identified in OCA patients of different ethnicity. The p.Arg217Gln mutation was detected in Caucasians from USA, Canada, and Northern-Europe; the p.Arg402* in Caucasians from Lebanon; the c.1037−7T>A in Caucasians and Japanese; the c.1366+4A>G in Caucasians; and the c.346C>T in Caucasians, Japanese, and Germans [22, 29]. The frequency of pathogenic mutations differs in different populations, and, therefore, these populations might vary in their genetic susceptibility to certain diseases. Based on our results and the results of previous studies, the identified pathogenic *TYR* mutations are not specific to the Hungarian population, as they have been detected worldwide in OCA patients [22, 29].

No pathogenic *OCA2* mutation was identified in the examined Hungarian individuals, although one patient carried the common p.Arg305Trp polymorphism in heterozygous form. This variant has been associated with human eye color and might be an inherited biomarker of cutaneous cancer risk [28, 30].

Three mutation and a common polymorphisms were detected on the *SLC45A2* gene. Two patients carried two heterozygous variants of the *SLC45A2* gene previously described by our workgroup: the p.Gly409Asp missense and the p.Gln487* nonsense mutations [31]. Both mutations are situated in transmembrane domains of the MATP protein (Uniprot: Q9UMX9). The locations of the mutations suggest that they impair the transport function of the MATP protein. MATP dysfunction might cause an acidic melanosomal lumen, leading to incorrect incorporation of copper into tyrosinase. The reduced tyrosinase activity could lead to the development of the OCA phenotype [32]. Besides, the p.Leu374Phe polymorphism was detected in nine Hungarian OCA patients. This variant has a striking population distribution, exists almost exclusively in Europeans, and has also been implicated in the development of different shades of hair, skin, and eye color [33].

Our results emphasize the importance of the parallel analysis of multiple genes for studying disease phenotypes. The OCA cases presented in this study and many other cases reported in the literature call our attention to the fact that clinical symptoms—which may overlap in many cases—are not sufficient for a diagnosis of different OCA forms: the molecular genetic investigation of all OCA genes is required to determine the subtype of the disease. The Hungarian OCA patients in this study exhibited identical clinical features (Table 1); however, molecular genetic investigation identified the OCA1 subtype in eight cases, the OCA4 subtype in two cases, and the molecular diagnosis was not clearly defined in three patients (Table 2). Even with these data, the genetic basis of the disease in seven patients carrying only one pathogenic *TYR* or *SLC45A2* mutation is still not completely explained. We wish to emphasize the screening of the non-pathogenic variants, which alone could not lead to the development of OCA, should be carried out in association with pathogenic variants that might have clinical significance. Further targeted sequencing of the genes involved in the syndromic OCA variants, including HPS and CHS, as well as genes involved in human pigmentation, is hoped elucidate the underlying disease-causing variant(s) [22].

Our results and previously reported studies suggest that, among the investigated genes, the majority of the mutations are located within the *TYR* gene [3, 4]. This result correlates well with the results obtained in other populations, as *TYR* mutations are the most common for OCA worldwide [1]. Screening of the *TYR* gene is, therefore, of primary importance for diagnostics. Mutations in the investigated Hungarian and in other previously reported OCA patients were found most frequently in exons 1 and 4 of the *TYR* gene [4]. In light of the fact that the majority of the identified *TYR* mutations are located within exon 1 and 4, we recommend screening these exons first.

According to our current knowledge, 10–25% of the isolated and syndromic OCA cases are not explained by paired, trans-oriented mutations in known genes [34, 35]. Based on our results and the results of previous studies [22], we suggest that screening non-Mendelian OCA-associated genes might elucidate the causative genetic variant for these cases.

## Conclusions

The genetic heterogeneity of OCA is extremely complex: both rare mutations of Mendelian genes and common variants of non-Mendelian genes can contribute to the development of the disease. Our multi-gene study provides novel data for the genetic diversity of OCA in Hungarians and indicates that approaches that take this complexity into account, including large-scale studies, are needed to complete our understanding of the genetic heterogeneity of this disease.

## Abbreviations

OCA: oculocutaneous albinism
OCA1: oculocutaneous albinism type 1
OCA2: oculocutaneous albinism type 2
OCA3: oculocutaneous albinism type 3
OCA4: oculocutaneous albinism type 4
OCA5: oculocutaneous albinism type 5
OCA6: oculocutaneous albinism type 6
OCA7: oculocutaneous albinism type 7
*TYR*: tyrosinase gene
*OCA2*: oculocutaneous albinism two gene
*TYRP*: tyrosinase-related protein gene
*SLC45A2*: solute carrier family 45, member 2 gene
*SLC24A5*: sodium/calcium/potassium exchanger 5 gene
*C10ORF11*: chromosome 10 open reading frame 11 gene
HPS: Hermansky–Pudlak syndrome
CHS: Chediak–Higashi syndrome
OA1: ocular albinism
*GPR143*: G protein-coupled receptor 143 gene
*TYRL*: tyrosinase-like gene
MATP: membrane-associated transport protein
